# Targeting protein synthesis in cancer cells

**DOI:** 10.18632/oncoscience.63

**Published:** 2014-07-04

**Authors:** Yvan Martineau, David Müller, Stéphane Pyronnet

**Affiliations:** INSERM UMR-1037, Université de Toulouse, Equipe Labellisée Ligue Contre le Cancer, Laboratoire d'Excellence Toulouse Cancer: TOUCAN, Toulouse, France

**Figure 1:**
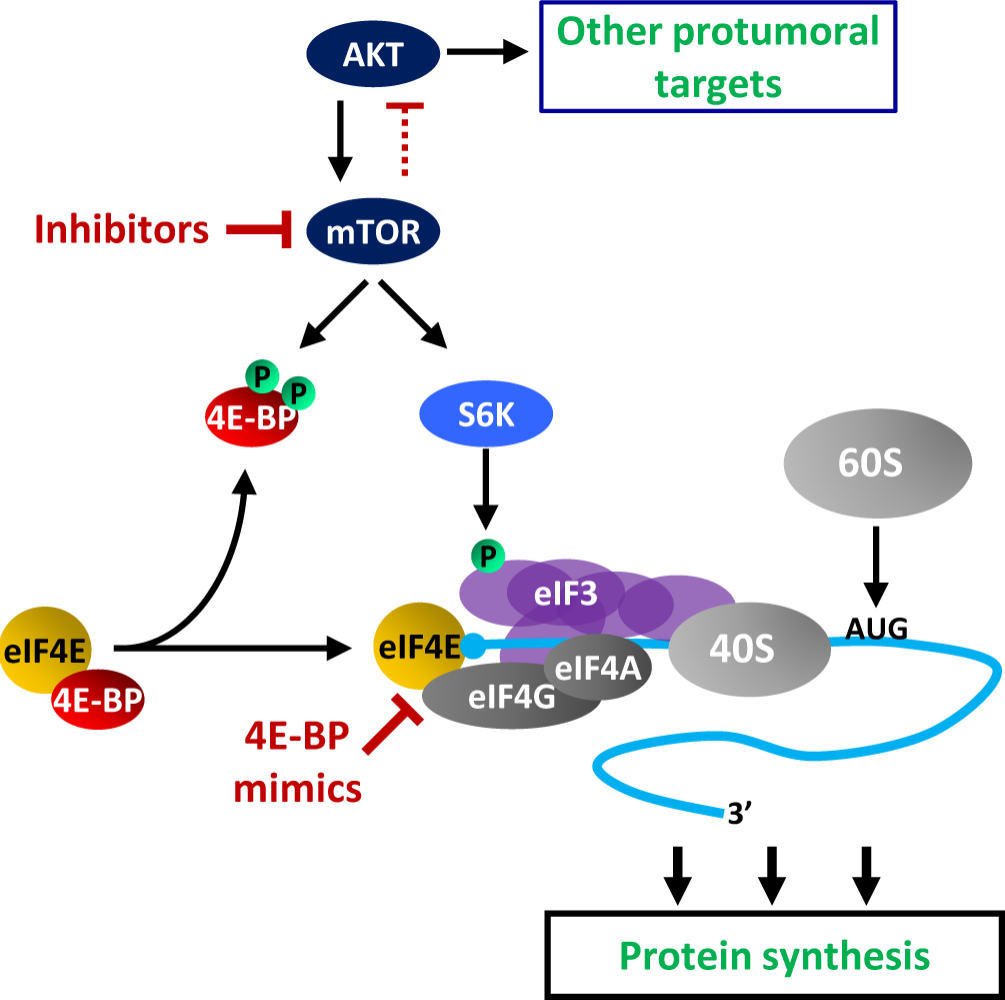
4E-BP mimics as an alternative to mTOR inhibitors

The G1 and S phases of the mitotic cell cycle normally insure that the parental cell attains a sufficient mass so as each daughter cell will have a size identical to that of the parental cell. This implies that before division cells must double their protein content, a process achieved through increase in protein synthesis. The most regulated step of protein synthesis is the initiation of mRNA translation into protein. Ribosome recruitment at the mRNA 5′ end is actually controlled by the mRNA 5′ cap binding protein eIF4E (eukaryotic translation initiation factor 4E) whose activity is inhibited by the hypophosphorylated forms of 4E-BPs (eIF4E-binding proteins 1 and 2) [1]. Upon mitogenic stimuli, mTOR phosphorylates 4E-BP1 and 4E-BP2 and the protein kinases S6K1 and S6K2 (S6Ks). 4E-BPs phosphorylation releases eIF4E which can interact with eIF4G itself bound to the RNA helicase eIF4A and to eIF3. Activated S6Ks phosphorylate eIF3 [2] and translation initiation is enhanced via eIF3-dependent recruitment of the 40S small ribosomal subunit and its subsequent joining with the large 60S subunit at the AUG initiator codon (Fig. 1).

Consistent with a role of mTOR in G1-S transition, mTOR inhibitors have been promising in the treatment of various cancers. However, although they yielded encouraging results in certain tumors, they remained often disappointing including in pancreatic cancer [3]. These failures can be explained at least in part by the loss of an mTOR-dependent feedback loop which normally restrains AKT activity (Fig. 1, red dotted inhibitory arrow, indirect effect). Indeed, upon pharmacological inhibition of mTOR, such feedback no longer exists and sustained AKT activity elicits other protumoral targets.

Another mechanism which can account for the inefficacy of mTOR inhibitors in pancreatic cancer has been recently highlighted. Although 4E-BP1 is highly expressed in the exocrine pancreas, we have actually found that a primary resistance to mTOR inhibitors exists in pancreatic cancer cells due to the dramatic downregulation of 4E-BP1 expression that accompanies pancreatic cell carcinogenesis. 4E-BP2 is poorly expressed in normal and cancer cells of the pancreas. The absence of 4E-BP1 and 4E-BP2 in pancreatic cancer cells prevents G1 phase inhibition by mTOR inhibitors owing to a less effective repression of general protein synthesis and, more specifically, to a lack in the repression of cyclin D1 post-transcriptional expression [4]. Consistently, the eIF4E/4E-BPs ratio is inversely correlated to the efficacy of mTOR inhibitors, and an acquired resistance to mTOR inhibitors occurs progressively when cells are chronically exposed to sub-lethal concentrations of mTOR inhibitors due to downregulation of 4E-BPs expression [5].

One alternative in targeting protein synthesis for the treatment of tumors resistant to mTOR inhibitors is to act on the pathway downstream of mTOR. This option can be envisioned for cancer cells lacking the mTOR targets 4E-BP1 and 4E-BP2 (due to either a primary or an acquired loss of expression, see above). In these cases, mimicking 4E-BPs' function independently of mTOR manipulation would have the double advantage of blocking protein synthesis while maintaining the negative feedback loop on other AKT-dependent protumoral pathways (Fig. 1). In support of this hypothesis, we have obtained encouraging data at least in cultured pancreatic cancer cells, where one 4E-BP mimic (4E2RCat) efficiently blocked protein synthesis and cell proliferation independently of 4E-BPs levels, while the effects of mTOR inhibitors remained dependent on sufficient 4E-BPs intracellular amounts [4]. 4E2RCat has been isolated by the group of Jerry Pelletier after screening of compounds preventing eIF4E interaction with eIF4G and therefore blocking protein synthesis (Fig. 1). Another related 4E-BP mimic (4E1RCat) has been shown by the same group to efficiently reverse chemoresistance in a mouse lymphoma model [6]. The need for alternatives to mTOR inhibitors in lymphoma is further supported by a recent paper indicating that not all lymphoma-derived cell lines express 4E-BP1 and that lymphoma cells lacking 4E-BP1 are resistant to mTOR inhibitors [7].

Thus, the “addiction” of cancer cells to protein synthesis appears as a druggable vulnerability which merits further investigations. This is particularly true for pancreatic tumors expressing very low levels of 4E-BP1 and 4E-BP2 and which are resistant to mTOR inhibitors. In a near future, the combination of 4E-BPs mimics with conventional chemotherapies may provide therapeutic interests.
